# Physical Separation of Amphiprotic-Polar AproticS for Simultaneous Extraction and Clean-up of Clomiphene from Plasma before Liquid Chromatographic Analysis

**DOI:** 10.22037/ijpr.2019.1100771

**Published:** 2019

**Authors:** Farhad Ahmadi, Nilofar Rahmani

**Affiliations:** a *Razi Drug Research Center, Iran University of Medical Sciences, Tehran, Iran.*; b *Department of Medicinal Chemistry, School of Pharmacy-International Campus, Iran University of Medical Sciences, Tehran, Iran. *; c *Department of Medicinal Chemistry, School of Pharmacy, Kermanshah University of Medical Sciences, Kermanshah, Islamic Republic of Iran.*

**Keywords:** Clomiphene citrate, Two phase freezing method, Liquid-liquid extraction, Doping control, Plasma extraction

## Abstract

An efficient and quantitative two phase freezing (TPF) method coupled with high performance liquid chromatography and UV-Vis detector was developed for the extraction, clean up, and determination of clomiphene citrate (CLC) in plasma samples. The separation of two miscible solvents by TPF method permits that the CLC was efficiently removed from proteins and transferred into the relative aprotic dipolar organic phase and in consequence, gave a higher recovery. The TPF method was compared to conventional liquid-liquid extraction and it gave more clean solution with better reproducibility. Linear range, limit of detection, and limit of quantification for CLC in plasma were obtained in the range of 0.06-18, 0.02, and 0.06 µg mL^-1^, respectively. The intraday and interday reproducibility for concentration of 1.0 µg mL^-1^ (%RSD) were 3.2% and 4.6%, respectively. In addition, the trueness, ruggedness, and reality of TPF were assessment. Finally, several real plasma samples were successfully analyzed using the developed method.

## Introduction

Clomiphene Citrate (CLC), 2-[4-(2-Chloro–1, 2–diphenylethenyl) phenoxy]-N, N-diethyl ethanamine citrate, is a synthetic estrogen-receptor modulator, and has the teratogenic effect similar to diethylstilbestrol and tomoxifen (1). The CLC is primarily use for treatment of women with absent or irregular ovulation and also used to increases follicular development in women receiving *in-vitro* fertilization (IVF) (2, 3). Unfortunately, the CLC misused as doping drug for stimulates endogenous androgen production and/or to overcome steroid-related side effects such as gynecomastia and therefore, appears in the list of World Anti-Doping Agency (4). The CLC tablets have geometric isomers cis and trans with ratio of 62% to 38%, respectively (5). Like the other drugs, CLC has a series of side effects such as breast pain, hot flashes, enlarged ovaries, severe abdominal pain, changes in vision, and allergic reactions (6). 

Additionally, several reports existed that a numbers of treatments with CLC were failed (2, 7-10). An important factor that is critical in doping control and/or on CLC efficiency is the plasma concentration of CLC (2). So, the identification and individualization of CLC concentration in plasma is the subject of many researchers in anti-doping and/or drug development laboratories which introduced a lot of selective analytical methods. According to our library studies these analytical methods for measuring CLC concentration in plasma are mostly based on chromatographic methods such as: HPLC-UV (11), HPLC-fluorescence (12, 13), LC-MS (14-18), LC-MS-MS (19), and potentiometric sensor (20). Mostly, these methods used the liquid-liquid extraction (12, 13, 15, 16, 18, 19) or dilution into 96 well plate (14) for protein precipitant and extraction of CLC into the organic phase of methyl tertiary butyl ether (MTBE).

Hage and Sengupta (21) reported that both the isomers of CLC have 1:1 interactions at a common binding region on human serum albumin (HSA) with an association constant of 7.5×10^6^ M^-1^ for cis and 1.3×10^6^ M^-1^ for trans forms. In addition, the CLC has a Log *P *_(octanol/water) _=6.7 and its solubility in non-polar organic solvent is weak and even insoluble in ether (22). 

Although the liquid-liquid extraction (LLE) is often used for the separation of CLC from plasma and urine samples, from the above points of view methyl tertiary butyl ether (MTBE) could not be able to completely precipitate all proteins and completely transfer the CLC from plasma into the organic phase. Therefore, an amount of CLC might be removed by HSA in extraction step and it caused low recovery and reproducibility.

Therefore, the previously reported data for the determination of CLC were not merit (23-25), so, Crewe *et al*. (18), introduced an optimized LLE method followed by LC-MS for the determination of CLC in plasma with coefficient of variations less than 10%. However, the presence of endogenous interfering peaks in some blank plasma and patient samples was reported as the main problem of their work and their attempts to resolve these peaks were broken and often resulted in long retention times. 

The low recovery, high RSD, and presence of interference peaks in the previous reports may be due to both interaction of CLC with HAS protein and to the presence of systematic error in LLE method that caused an increased risk of analyte losses and higher %RSD (14). 

Because of the shortcomings and disadvantages of LLE method, the miscible organic solvents such as methanol and/or acetonitrile are widely used to produce a homogeneous solution for complete precipitation of proteins (26). Additionally, due to greater contact between the extracting miscible organic liquid and plasma matrix the analyte target may be completely removed and extracted into the liquid phase. In this method the extracting solution (contain both organic and aqueous phase) is centrifuged and supernatant directly injected into the analytical apparatus. However, as the extracting solution is a binary mixture of aqueous and non-aqueous solvents therefore, some of aqueous soluble proteins are transferred into the extracting solution and give strong interference peaks. In addition, these proteins adsorbed the CLC in extracting solution and decreased the free concentration of CLC. To overcome this problem, the separation of both aqueous and non-aqueous solvents in homogeneous extracting solution is necessary. In this work we introduced a simple, sensitive, and reproducible method for simultaneous clean-up and extraction of CLC from plasma using two phase freezing (TPF) method in which the organic phase is separated from the aqueous solution and a clear solution with lower interference and higher CLC is obtained. The efficiency of the TPF method may depends on the extraction conditions such as pH of solution, type of organic solvent, the volumetric ratio of organic to aqueous solvent, and extraction cycles, therefore, these parameters were optimized. Our results demonstrated that the TPF method solved the protein binding problem of CLC and provided a clear solution with higher recovery in one step that allows improving the sensitivity and higher purities.

## Experimental

*Chemical reagents*

All organic solvents such as methyl tertiary butyl ether (MTBE), methanol (MeOH), ethanol (EtOH), acetonitrile (ACN), propanol (Pro), and other chemical reagents such as acetic acid (CH_3_COOH), ortho-boric acid (H_3_BO_3_), hydrochloric acid (HCl), phosphoric acid (H_3_PO_4_), ammonia (NH_3_), sodium hydroxide (NaOH), were analytical grade and purchased from Merck (Darmstadt, Germany). Briton Robinson buffer solution (0.1 mol L^−1^) was prepared by dissolving 1.236 g of H_3_BO_3_, 1013 μL of H_3_PO_4_, and 857 μL of CH_3_COOH in 500 mL of double distilled water. Clomiphene citrate (CLC) was purchased from Sigma–Aldrich (St. Louis, MO, USA). The stock solution of CLC was prepared by dissolving 59.8 mg in 10 mL of ACN and stored at 0.0 °C. The working solution was prepared by diluting an appropriate volume of stock solution with ACN. One plasma sample was obtained from a healthy volunteer from Imam Reza hospital (Kermanshah, Iran). The real plasma samples were obtained from the patients who are treated with the CLC, and then stored at −80 °C until use. 

*Apparatus*

All high performance liquid chromatography (HPLC) analyses were carried out by a KNAUER liquid chromatography system equipped with a manual six valve injector fitted with a 20 μL loop (7725i- made in USA) a PDA detector (UV detector 2600 model) and a temperature controller (Jet stream two plus). The apparatus was controlled by EZ-Chrome Elite software. The separation of CLC was performed at 40 °C on a Eurospher RP-C18 column (250×4.6 mm i.d., Europher 100–5 C18) containing sorbent with particle size of 5 μm and pour size of 100 Å that equipped with a guard column (Europher 100–5 C18). A mobile phase containing MeOH: ACN: phosphate buffer (pH = 2.5) (30: 30: 40 V/V %) with flow rate of 1.5 mL min^−1^ was used for elution of analyte. The detection wavelength that gives the maximum sensitivity was 240 nm. Under these chromatographic conditions the CLC was eluted in 3.25 min. A Sartorius electronic balance (TE124S, Germany) was used for weighting samples. The pH of solutions was measured by a Metrohm pH meter (827 pH lab model). The separation of ACN from the homogeneous extracting solution by TPF method was performed by refrigerator model Ang-platilab 500 at -40 °C. A Hettich centrifuge model EBA20 was used for protein precipitation. 

*Extraction and clean-up of CLC by two phase freezing method*

Into 1.0 mL of plasma solution 0.2 mL of Briton Robinson (BR) buffer (pH = 3.5, 0.075 mol L^-1^), and 0.3 mL of ACN was added to the mixture to achieve the final volume of 1.5 mL, then vigorously shaken for 5 min and the solution was centrifuged at 6000 rpm for 5 min. After the proteins were precipitated and two phases (extracting solution and proteins) had become separated, the liquid phase was carefully removed using a pipette. The volumes of both extracting solution and bottom phases were recorded. For separation of ACN from the extracting solution by TPF method, an appropriate KCl solution (1.5 mol L^-1^) was added to the extracting solution that produced a final concentration of 1 %W/V of KCl and then placed in refrigerator at −40 °C for 5.0 min. The tube was centrifuged at −20 °C by a universal centrifuge (model PIT 320R) to separate the ACN from the frozen aqueous phase and the ACN was removed by pipette from the aqueous frozen, collected and evaporated under N_2_ gas at 50 °C. The remained residue was dissolved in 50 μL of ACN, and 20 μL was injected into the HPLC system.

*LLE extraction by MTBE*

The LLE extraction was performed according to the literature (13) with some minor modification as follows: to 0.5 mL of plasma, 20 µL aqueous solution containing 50 ng of CLC and 2.5 mL of MTBE was added and the samples were mixed for 5 min. The solution was centrifuged, the organic phase was removed, then evaporated to dryness and finally the residue was dissolved in 100 µL of MTBE. Then 20 µL of the resulting solution was injected into the HPLC system.

*Method validation*

The method validation was performed via evaluation of linearity, intra- and inter-day precision, limit of detection (LOD) and limit of quantification (LOQ). The utilized validation evaluation was performed on one blank plasma sample (age of 20 years). The calibration curve of each plasma sample was constructed with 8 concentration levels in the range 0.06 to 18 μg mL^-1^. The extraction and clean-up was performed according to section 2.3. The chromatogram area of each drug was plotted versus the concentration of each spiked drug. The 3.0 replicate analyses for each level were performed. 

The trueness (absolute recovery) of the method was evaluated on 1.0 mL of three plasma samples of healthy volunteers , spiked with two different concentration level of CLC (0.5 and 1 μg mL^−1^) under optimum conditions. 

*Ruggedness and reality of TPF method*

For ruggedness evaluation of the TPF method, the RSD results of intraday assay of two different analysts were compared. The experiments were performed in the same laboratory on the same plasma samples. Furthermore, a Korean HPLC Young Lin (YL9100) with the (Macherey Nagel, 100-5 C18) column was used for measuring CLC in the spiked plasma series in order to show the reality of the method’s performance in daily routine applications.

## Results and Discussion

*HPLC conditions and optimum detection wavelength*

The CLC is a weak polar compound with pKa= 9.3 and weak basic properties, therefore, in neutral conditions and at reversed phase column it is separated in long retention times. So, in order to have a good separation on the C-18 column with a reasonable capacity factor (*k*′) the pH of mobile phase was considered as a critical point for changing the CLC to protonated form with positive charge in order to be eluted at a suitable retention time. In this work various mobile phases were evaluated with isocratic elution such as:

ACN: MeOH: phosphoric acid (0.01 mol L^−1^; pH = 2.5) with 20: 20:60 V/V%. 

ACN: MeOH: H_2_O: phosphoric acid (0.01 mol L^-1^; pH = 2.5) with 20: 20: 20: 40 V/V%.

ACN: MeOH: phosphoric acid (0.01 mol L^-1^; pH = 2.5) with 30: 30: 40 V/V%.

ACN: phosphoric acid (0.01 mol L^−1^; pH = 2.5) 60: 40 V/V%.

The results revealed that when the mobile phase (III) was used the elution of CLC on the Eurospher RP-C18 column give satisfactory* K*′ value. The CLC has two typical maximum adsorption peaks at 254 nm and 298 nm. In this work various wavelengths, i.e. 235, 240, 245, 254, and 298 nm were tested to monitor the optimum wavelength that was measured via response factor of the detector. It was found that the detector response factor at 240 nm was the maximum and therefore 240 nm was selected as optimum detection wavelength for CLC determination.

*Optimization of TPF method*

However, in order to obtain the maximum extraction and high reproducibility by TPF method for detection of CLC in plasma samples, several important variables, having effect on TPF efficiency such as pH of solution, type and volume of miscible organic solvent, the ratio volume of organic to aqueous solvent, extraction cycles, and salting out were optimized in binary mixture of ACN: aqueous buffer solution. The percent extraction yield (%EY) was considered as a response for each optimization parameter.

Extraction factor (EF) is defined as the ratio of the CLC concentration in the ACN phase (C_ACN_) to the initial concentration of CLC (C_0_) within the aqueous solution:

Equ.1 $$\mathrm{EF}=\frac{C_{\mathrm{ACN}}}{C_{0}}$$

The C_ACN _was obtained from the calibration graph. 

Percent extraction yield (%EY) is defined as the percentage of the ratio of CLC amount extracted into the ACN phase (n_ACN_) and the total CLC amount (n_0_) in aqueous sample (Eq. 2). 

Equ.2 $$\mathrm{\%E}Y=\frac{n_{\mathrm{ACN}}}{n_{0}}\times 100=\frac{C_{\mathrm{ACN}}\times V_{\mathrm{ACN}}}{C_{0}\times V_{\mathrm{aq}}}\times 100=\mathrm{EF}\times \frac{V_{\mathrm{ACN}}}{V_{\mathrm{aq}}}\times 100$$

Where V_ACN_ and V_aq_ are the volumes of ACN phase and aqueous solution, respectively.

*Effect of pH of aqueous solution*

**Figure. 1:**
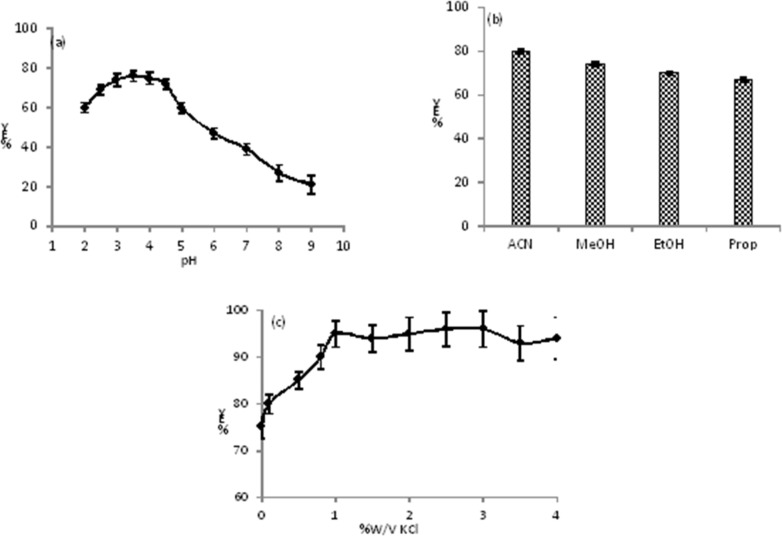
**(a)** Extraction yield (%EY) of CLC into the ACN versus pH of aqueous phase, the conditions were as follows: V_aq_= 1.0 mL, V_ACN_=0.3 mL, the amount of CLC=1.0 μg mL^−1^, V_buff_= 0.2 mL, number of extraction cycle = 1, no salt was added; **(b)** the effect of various organic phase on extraction yield (%EY) of CLC, the conditions were as follows: V_aq_=1.0 mL, V_org_= 0.3 mL, the amount of CLC =1.0 μg mL^−1^, V_buff_=0.2 mL, number the amount of CLC=1.0 μg mL^−1^, pH aqueous phase =3.5, number of extraction cycle=1, no salt was added; **(c)** effect of salting out on extraction efficiency, the conditions were as follows: V_aq_=1.0 mL, V_ACN_=0.3 mL, V_buff _= 0.2 mL, the amount of CLC=1.0 μg mL^−1^, pH aqueous phase = 3.5, number of extraction cycle = 1. All measurements were three times repeated (n = 3)

The is an amine weak base with pKa = 9.3, and may has different structures and charges in various pHs (27). Therefore, the pH has an important role on the extraction of CLC into the ACN. To examine the effect of pH on the extraction efficiency, the extraction yield of CLC was studied at specific concentration of the CLC (1 μg mL^-1^) in the pH range of 1.5 to 11.0 (pH was adjusted by BR buffer). The results showed that the changes of peak area in the pH range of 2.5 to 4.5 was roughly constant while a noteworthy fluctuation in the peak area* vs. *pH took place below and above the stated pH limits (Figure 1a). Under strong acidic pHs (pH < 2.5) the CLC may be unstable and decomposed. We previously reported that the ACN has basic properties (28, 29) and in the pH range of 2.5-4.5 the type III amine of CLC is protonated and has acidic properties therefore, an acid-base interaction was occurred between CLC and ACN molecules. At higher pHs (pH > 4.5) the concentration of proton (H^+^) in the solution decreased, so more H_2_O molecules interact to lone pair electron of amine of CLC via hydrogen binding. Therefore, the tendency of CLC to the ACN reduced and the extraction decreased. In the pH range of 2.5 to 3.5 the extraction yielded slowly increased and reached to its maximum value at 3.5 and then a steep decrease was observed with pH = 4.5. So, we have selected the pH = 3.5 for the next studies.

*Choice of organic solvent on extraction and extraction cycles*

The extraction of target into the organic solvent must be affected by the solvent medium and depends on a range of factors including: type of solvent, solvent donor ability, solvent acceptor ability, and solvent-solute hydrogen-bonding properties. During the TPF extraction in homogenous binary mixture solution, the organic solvent should remove the CLC from proteins and also replace the H_2_O molecules of first solvation shell of CLC with its own molecules. The effect of various miscible organic solvents such as MeOH, EtOH, ACN, and propanol on extraction efficiency was intensively investigated for specific concentration of the CLC (1 μg mL^-1^) in the pH = 3.5. In our investigation, the maximum extraction efficiency was obtained for ACN in pH = 3.5 (see Figure 1b).

The water molecules have high solvating (DN = 33) and hydrogen-bonding ability, while the ACN is an aprotic and protophobic dipolar solvent and rarely interacts with CLC by hydrogen bonding (DN = 14.1) in neutral and or alkaline pHs (29). Therefore, in pH = 3.5 due to hydrophobic and acid-base interactions between ACN and protonated CLC, the highest extraction efficiency were obtained in comparison with other organic solvents. Due to high miscibility of ACN with plasma and proteins denaturation, the CLC is completely removed. Also highest interaction occur between ACN and CLC and caused more extraction efficiency. 

*Salting out effect and cycle of extraction*

The effect of salting-out on TPF efficiency was investigated using KCl in the range of 0.0 to 4.0 %W/V at pH = 3.5. At above 0.0 %W/V of KCl, an increasing in the extraction efficiency of CLC in ACN phase was observed and reached to a constant value at 1.0 %W/V, therefore, 1.0 %W/V of KCl was used (see Figure 1c). We studied the effect of number of extracting cycle and this factor does not have any effect on extraction yield. In addition, the best solvent ratio (aqueous solution/ACN) of 3 with one extraction cycle gave highest recovery (more than 93%) and reproducibility. 

*Comparison of TPF with LLE method in plasma samples*

To our search data we achieved the proposed TPF gave less concentration factor (aqueous/CAN = 3) in comparison to LLE method which used MTBE as extracting solvent (12, 13, 15, 16, 18, 19). Nevertheless, the LLE method has: environmental and health problems and low reproducibility. In addition, due to inherent immiscibility of MTBE with plasma, no well interaction occurred between CLC molecules in plasma and organic solvent molecules. The results of proposed method and LLE method were placed in Table 1.

In laboratory by addition of MTBE and/or ACN into plasma the proteins were precipitated and produced solutions with three phases (protein + aqueous + MTBE) in LLE and two phases (protein + binary mixture of aqueous-ACN solution) for TPF. The LLE is carried out in one step including: addition of MTBE → vigorously shaking → separation of three phases → collection of organic phase → direct injection of organic phase into the chromatographic system; while TPF method is performed in two steps including: I) addition of ACN → vigorously shaking → separation of two phase; II) separation of ACN from the homogenous solution by TPF method → injection of ACN into the chromatographic system. To compare the two methods the %EY for each method was measured as follows:

For TPF in plasma sample the extraction factor (EF_pla_) is defined as the ratio of CLC concentration in the ACN phase (C_ACN_) to the initial concentration of CLC (C_HBS_) within the homogeneous binary solution (Eq. 3):

Equ.3 $${\mathrm{EF}}_{\mathrm{TPF}}=\frac{C_{\mathrm{ACN}}}{C_{\mathrm{HBS}}}$$

The C_ACN _was obtained from the calibration graph similar to equation (1). 

The percent extraction yield of TPF method (%EY_TPF_) is defined as percent ratio of amount of CLC extracted into the homogeneous binary solution phase (n_HBS_) to total amount of CLC (n_P_) in plasma sample (Eq. 4). 

Equ.4 $$\%{\mathrm{EY}}_{\mathrm{TPF}}=\frac{n_{\mathrm{HBS}}}{n_{P}}\times 100=\frac{C_{\mathrm{HBS}}\times V_{\mathrm{HBS}}}{C_{P}\times V_{T}}\times 100$$

Where V_HBS_ and V_T_ are the volumes of homogeneous binary solution phase and total volume sample, respectively.

The V_T_ and V_HBS_ were measured by equations 5 and 6, respectively.

 Equ.5 $$V_{T}=V_{\mathrm{pro}}+V_{\mathrm{aq}}+V_{\mathrm{ACN}}$$

Equ.6 $$V_{\mathrm{HBS}}=V_{\mathrm{aq}}+V_{\mathrm{ACN}}$$

Where the V_pro_ is the volume of precipitated protein.

By inserting equation (3) into the equation (4) the equation (7) is obtained:

Equ.7 $${\mathrm{\%EY}}_{\mathrm{TPF}}=\frac{C_{\mathrm{ACN}}\times V_{\mathrm{HBS}}}{{\mathrm{EF}}_{\mathrm{TPF}}\times C_{P}\times V_{T}}$$

The V_HBS_ is measured as: $V_{\mathrm{HBS}}=V_{T}-V_{\mathrm{pro}}$.

The %EY_LLE_ for liquid-liquid extraction was measured by equation (8) as follows:

Equ.8 $$\%{\mathrm{EY}}_{\mathrm{LLE}}=\frac{C_{\mathrm{MTBE}}\times V_{\mathrm{MTBE}}}{C_{P}\times V_{P}}\times 100$$

Where V_P_ and V_MTBE_ are the volume of plasma and MTBE, respectively. As it is observed in Table 1, the TPF method gave better extraction yield.

*The advantageous of TPF than to other phase separation methods*

 ACN and water are completely miscible at any ratio and the phase separation of this binary mixture (ACN-water) was often performed by addition of an inorganic salt. At pure ACN high polar compounds and proteins poorly soluble, while in binary mixture of ACN-water solution these compounds are significantly soluble (30); in other words, the water molecules in the solution help to enhance solubility of the proteins in ACN. So, we believe these protein molecules in binary mixture solution act as a barrier to complete extraction of CLC and also reduced the life time and efficiency of chromatographic column. Therefore, the phase separation seems to be critical. The common protocol for phase separation of ACN-water mixture is salt addition, in which a water-enriched lower and an ACN-enriched upper phase are generated. Although, magnesium sulfate is often used than other salts like sodium sulfate or sodium chloride (31) for phase separation of ACN, it may give a very poor recovery for drugs due to metal complex formation. Already, the complex formation between magnesium ions and tetracyclines and quinolones were reported (32). Additionally, due to non-volatile properties of magnesium sulfate it may accumulate in the LC–MS interface (33).

**Figure 2:**
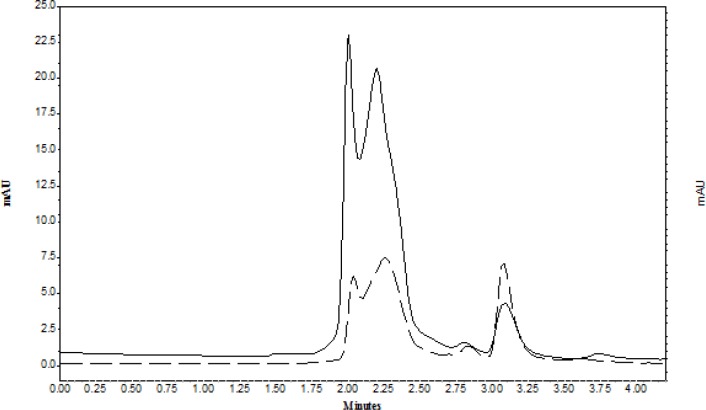
A sample chromatogram of CLC that extract from plasma in: (solid line) in supernatant of water-ACN before TPF; (dash line) after TPF; under optimum conditions, the amount of CLC was 0.1 µg mL-1).

As the complex formation of analytes with magnesium sulfate caused insufficient recoveries, therefore, various phase separation methods such as: counter current salting-out homogenous liquid–liquid extraction (34), salting out supported liquid extraction (SOSLE) (32), and LLE (19) were used for extraction of organic analyte from plasma and biological fluids. However, these methods were performed in several steps and caused loss of analyte. To overcome this problem the physical phase separation seems to give higher recovery and clear solution (35). Also, the recovery and reproducibility of the work is very important. Figure 2 represented the chromatogram of CLC in supernatant of water-ACN before TPF (solid line) and after TPF (dash line). As it is observed in TPF the peak area of CLC increased and the peak area of proteins significantly reduced.

*Validation of the method*

The validity, reproducibility, and clean satisfactory of solution in plasma samples were evaluated in terms of linear range (L.R), limits of detection (LOD), limits of quantification (LOQ), precision (RSD), trueness, ruggedness, and reality. 

**Figure 3:**
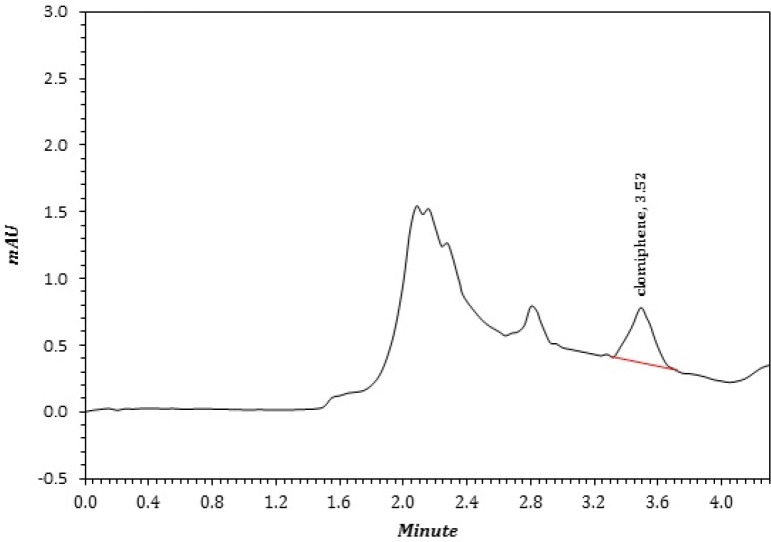
A sample chromatogram of CLC that extract by TPF method from a patient that extracted according to section 4.6 and under optimum conditions

**Table 1:** The comparison of TPF method with LLE

**Compound**	**0.5 µg mL** **-1**		**1.0 µg mL** **-1**	
	%EYTPF (n = 3)	%EYLLE (n = 3)	%EYTPF (n = 3)	%EYLLE (n = 3)
**CLC**	95.4±2.8	80.2±9.2	94.5±3.1	85.4±8.3

**Table 2:** the analytical validations of proposed method

**Compound**	**Linear Range (µg mL** **-1** **)**	**LOD (µg mL** **-1** **)**	**LOQ (µg mL** **-1** **)**	**%RSD(n=3)** **intraday**	**%RSD (n = 3)** **interday**	**%Absolute recovery (n = 3)**
CLC	0.06-18	0.02	0.06	3.2	4.6	93.5±3.1

**Table 3:** Five real samples that analyzed by proposed method

**NO of sample patient**	**Spiked (µg mL** **-1** **)**	**Found (µg mL** **-1** **)**
**1**	0.0	ND a
	0.5	0.46
	1.0	0.94
**2**	0.0	0.15
	0.5	0.61
	1.0	1.04
**3**	0.0	0.23
	0.5	0.79
	1.0	1.18
**4**	0.0	0.43
	0.5	1.05
	1.0	1.54
**5**	0.0	ND a
	0.5	0.54
	1.0	0.95

a not detect.

*Linear range (L.R), LOD, LOQ, precision and trueness*

Characteristics of the TPF method plasma-matched calibration curve were established using a plasma sample (20 years old) spiked with various amounts of CLC in the concentration range of 0.06 to 18 μg mL^-1^. Each concentration level was repeated three times (n = 3) and the mean peak area was considered for each corresponding concentration. Limit of detection (LOD) and limit of quantification (LOQ) for each plasma sample were calculated as: 3×S/N and 10×S/N, respectively. All data were shown in Table 2.

The precision of TPF method was evaluated based on intraday and interday repeatability for plasma samples that spiked with 1.0 µg mL^-1^ of CLC. The intraday repeatability study was carried out over one plasma sample (20 years old) spiked with 1.0 µg mL^-1^ of CLC (n = 3) on the same day and the interday was studied in three consecutive days (n = 3; total analysis=3×3). The data were shown as %RSD of the %EY, and placed in Table 2. 

For checking trueness of TPF method in real patients, the recovery of CLC was studied in one type of plasma sample (20 years old), spiked with one concentration levels of CLC. Absolute recovery (A.R) for 1.0 µg mL^-1^ was obtained by comparing mean peak area of CLC in plasma after the TPF procedure with relative peak areas of CLC before the TPF. The plasma sample before spike of CLC was analyzed and the CLC was not above the LOD of the method (Table 2).

*Ruggedness and reality studies*

The ruggedness of proposed method was evaluated by comparison of RSDs, obtained by two analysts in the same laboratory and the results demonstrated that none of them gave the RSD more than 5.0 %. 

Also, the reality of TPF method, evaluated by a different HPLC and column with one analyst and the two side t-test (degree of freedom = 5; *p* value < 0.05) revealed that this method is suitable for daily routine use (36).

*Interferences and additional peaks*

The interference effects of three drugs with most likely structure and/or co administrating with CLC such as Tomoxifen, letrozole, and human menopausal gonadotropin (HMG) were tested. The results revealed that under optimum conditions most of the tested interferences did not have a remarkable effect and also did not coelute with CLC. 

*Real sample application of the method*

In clinical usage of CLC, the concentration less than 0.1 µmol L^-1^ in plasma (25, 37) is considered as “sub therapeutic level (STL)” and has inappropriate clinical action. However, it must be mentioned that the enrichment factor (EF) of proposed method is 3 and the LOQ of method is equal to STL. Therefore, using less than the STL is commonly associated with a negative outcome. To overcome this problem in real samples, especially for doping control, the extracting ACN sample is manually evaporated and the residue was re-dissolved in 50 µL of ACN and re-analyzed for presence of CLC. In this manner the EF increased up to 16. Five patients were analyzed and the results were placed in Table 3. Figure 3 represented a real sample of CLC in plasma.

## Conclusion

The proposed TPF method is able to noteworthy removal of CLC from the proteins and extracts into the organic phase because of its well miscibility into the plasma. The phase separation induced by the freezing aqueous phase permits the use of an aprotic dipolar acceptor phase (ACN) to efficient removal and extract of CLC from the plasma phase. Furthermore, the TPF provides a higher clean solution containing the analyte, achieved by a single extraction and clean-up step. The clean-up capability of the method is superior to LLE and gave better analysis time and consumables cost. Additionally, due to presence of a higher concentration of ACN in the final extract solution it does not permit the presence of impurities and save the chromatographic column.
